# Maternal soluble urokinase plasminogen activator receptor levels in intrahepatic cholestasis of pregnancy: a predictor of neonatal intensive care unit admission

**DOI:** 10.1590/1806-9282.20251109

**Published:** 2025-12-05

**Authors:** Betül Akgün Aktaş, Zahid Agaoglu, Betül Nur Peker, Ezgi Başaran, Nazlı Orhan, Burcu Bozkurt Özdal, Dilek Şahin

**Affiliations:** 1 Uşak Education and Research Hospital, Department of Obstetrics and Gynecology, Division of Perinatology – Uşak, Turkey.; 2 Ministry of Health, Ankara City Hospital, Department of Obstetrics and Gynecology, Division of Perinatology – Ankara, Turkey.

**Keywords:** ICP, NICU, Receptors, Urokinase plasminogen activator, Pregnancy outcome

## Abstract

**OBJECTIVE:**

The aim of this study was to compare maternal plasma soluble urokinase plasminogen activator receptor levels in pregnant women diagnosed with intrahepatic cholestasis of pregnancy with those in healthy pregnant women and to evaluate its predictive value for neonatal intensive care unit admission.

**METHODS:**

This study is a prospective case-control study and was conducted with a total of 80 participants, including 38 pregnant women with intrahepatic cholestasis of pregnancy and 42 healthy pregnant women between 28 and 37 weeks of gestation. To evaluate the predictive value of maternal suPAR and bile acid levels for neonatal intensive care unit admission, receiver operating characteristic curves were generated.

**RESULTS:**

Maternal serum alanine aminotransferase, aspartate aminotransferase, and direct bilirubin levels were statistically significantly higher in the intrahepatic cholestasis of pregnancy group compared to the control. In the intrahepatic cholestasis of pregnancy group, the plasma soluble urokinase plasminogen activator receptor level was 0.42±0.6 ng/mL, whereas in the control group it was 0.18±0.1 ng/mL (p=0.038). The intrahepatic cholestasis of pregnancy group delivered at an earlier gestational age and with lower birth weight, and the need for neonatal intensive care unit admission was statistically significantly higher. In both the severe and mild intrahepatic cholestasis of pregnancy groups, spontaneous preterm birth was more frequent than iatrogenic preterm birth. In the severe intrahepatic cholestasis of pregnancy group, there was one neonatal death and one meconium-stained birth. The discriminatory power of soluble urokinase plasminogen activator receptor levels in predicting neonatal intensive care unit need was found to be statistically significant (area under the curve: 0.757; 95%CI 0.552–0.962; p=0.022).

**CONCLUSION:**

High maternal plasma soluble urokinase plasminogen activator receptor levels may predict adverse pregnancy outcomes.

## INTRODUCTION

Intrahepatic cholestasis of pregnancy (ICP) is one of the most common liver disorders during pregnancy, typically associated with pruritus without rash, abnormal liver function, and elevated bile acid (BA) levels in the later gestational weeks^1^. Although the etiology of ICP remains unclear, it has been shown to be an inflammatory process associated with multiple pregnancies, advanced maternal age, and hormonal, genetic, and environmental factors^2,3^. ICP carries risks such as preterm birth, neonatal respiratory distress, meconium-stained amniotic fluid, and fetal death^4^.

Soluble urokinase plasminogen activator receptor (suPAR) is a marker that has been the subject of research in the diagnosis and prognosis of diseases accompanied by inflammation in the last decade and with the pandemic^5-7^. The system is a thrombolytic cascade that leads to fibrinolysis through the conversion of plasminogen to plasmin. Urokinase plasminogen activator receptor (uPAR) is a membrane receptor associated with endothelial cells, inflammatory blood cells, and growth factors seen in cancer, whose expression increases with inflammation and cytokines^8^. Upon activation of plasmin and other proteases, uPAR is cleaved into a soluble form and released into the circulation. SuPAR levels increase, especially in chronic inflammatory environments, and due to its stable form, it is less affected by acute changes and distinguishes itself from classical acute-phase reactants^9^. We aimed to measure suPAR levels in pregnant women with ICP, which is thought to be useful in predicting the course of disease in different diseases such as inflammation and cancer, and to examine the predictive value of suPAR in neonatal intensive care unit (NICU) admission.

## METHODS

This study was designed as a prospective case-control study and was approved by the Ethics Committee of the Ministry of Health Ankara Bilkent City Hospital, with confirmation of compliance with the Declaration of Helsinki (date: September 6, 2023; approval number: E2-23-4880). The study was conducted between January 1, 2024, and January 1, 2025, with a total of 80 participants, including 38 pregnant women diagnosed with ICP and 42 healthy pregnant women, all between 28 and 37 weeks of gestation, who presented to Ankara City Hospital. The diagnosis of ICP was based on the presence of pruritus, an elevated fasting serum BA level above 10 mmol/L, and elevated serum transaminases^1^. Pregnant women who did not give consent to participate; those with infectious or inflammatory comorbidities, multiple pregnancies, renal disease, preeclampsia, and diabetes; and those with fetal chromosomal or structural anomalies were excluded. The control group was randomly selected from participants. Informed consent was obtained from all participants. Demographic characteristics were recorded, and ultrasound screenings were performed. Liver function tests at the time of diagnosis and postnatal outcomes were included in the study. In addition, the ICP group was subdivided into mild and severe subgroups based on BA levels.

Venous blood samples were collected from participants in the ICP group on the day they were diagnosed with ICP and from the control group at a comparable gestational week. Peripheral venous blood samples were collected into K2EDTA tubes. Measurements were conducted using the Heales MB-580 fully automated enzyme-linked ımmunosorbent assay (ELISA) device with the Human suPAR ELISA kit (ELK Biotechnology, Catalog No: ELK9036). The kit's sensitivity was 0.056 ng/mL, with a detection range of 0.16–10 ng/mL. The intra- and inter-assay coefficients of variation (CVs) were <8 and <10%, respectively.

Statistical analysis was performed using IBM SPSS Statistics 26.0. The normality of the variables was tested using both the Shapiro-Wilk and Kolmogorov-Smirnov tests. Group comparisons were conducted using the Student's t-test. A p-value of <0.05 was considered statistically significant.

To evaluate the predictive value of maternal suPAR and BA levels for NICU, receiver operating characteristic (ROC) curves were generated, and area under the curve (AUC 95%CI) values were calculated. Sample size estimation was performed using G*Power software, with a 5% margin of error and 80% power, resulting in a minimum requirement of 27 patients per group^10^.

## RESULTS

Table 1 presents the baseline characteristics and obstetric outcomes of the study population. Demographic and obstetric features were similar between the ICP group and the healthy control group. The gestational age at diagnosis in the ICP group was 32.9±2.6 weeks, and the BA level was 43.9±31.5 mmol/L. Maternal serum ALT, AST, and direct bilirubin ­levels were significantly higher in the ICP group compared to the control group. The plasma suPAR level was 0.42±0.6 ng/mL in the ICP group, while it was 0.18±0.1 ng/mL in the control group (p=0.038). The ICP group delivered at an earlier gestational week and had lower birth weights. The need for NICU admission was significantly higher [n=9 (23.6%) in the ICP group vs. n=3 (7.1%) in the control group; p=0.024].

**Table 1:** Baseline characteristics and obstetric outcomes of the study population.

	Cholestasis group (n=38)	Control group (n=42)	p-value
Maternal age (years)	29.8±5.0	27.7±5.2	0.075
Nulliparity	16 (42.1)	15 (35.7)	0.558
Gestational week at diagnosis	32.9±2.6	34.1±2.9	0.079
SuPAR (ng/mL)*	0.42±0.6	0.18±0.1	**0.038**
ALT (U/L)*	190.2±216.8	16.7±7.8	**<0.001**
AST (U/L)*	130.1±169.4	16.5±9.2	**<0.001**
Total bilirubin (mg/dL)	0.66±0.3	0.49±0.1	0.089
Direct bilirubin (mg/dL)*	0.31±0.2	0.16±0.1	**0.021**
Gestational weeks at birth*	35.6±2.2	38.9±1.1	**<0.001**
Emergency cesarean	11 (28.9)	7 (16.6)	0.983
Birth weight (g)*	2,767.2±584.7	3,238.7±411.5	**<0.001**
APGAR fifth minute <7	2 (5.2)	0 (0)	**<0.001**
NICU*	9 (23.6)	3 (7.1)	**0.024**

Note: Data are presented as mean ± standard deviation, number (n), and percentage (%).

* Bold values denote statistical significance at the p<0.05 level. SuPAR: soluble urokinase plasminogen activator receptor; APGAR: appearance, pulse, grimace, activity, and respiration; ALT: alanine aminotransferase; AST: aspartate aminotransferase.

In Table 2, the ICP group was defined as mild ICP with a BA value below 40 mmol/L and severe ICP with a BA value of 40 mmol/L and above, and baseline characteristics are given. The baseline features were similar between the mild and severe ICP groups, but BA and suPAR levels were markedly higher in the severe group. In the severe ICP group, delivery occurred at 34.7±3.0 gestational weeks, compared to 36.3±1.1 weeks in the mild group (p=0.055). In both groups, spontaneous preterm labor was more common than iatrogenic.

**Table 2:** Baseline characteristics and obstetric outcomes of patients with mild and severe intrahepatic cholestasis of pregnancy.

	BA<40 (n=22)	BA≥40 (n=16)	p-value
Maternal age (years)	29.3±4.9	30.6±5.2	0.452
Nulliparity	9 (40.9)	7 (43.7)	0.861
Gestational weeks at diagnosis	33.4±2.0	32.3±3.2	0.185
Bile acid (mmol/L)*	20.5±8.0	75.3±22.4	**<0.001**
SuPAR (ng/mL)	0.33±0.3	0.54±1.1	0.337
ALT (U/L)*	105.6±85.5	306.5±284.2	**0.003**
AST (U/L)*	57.2±31	230.2±225.8	**0.001**
Total bilirubin (mg/dL)*	0.55±0.2	0.81±0.4	**0.024**
Direct bilirubin (mg/dL)*	0.22±0.1	0.43±0.2	**0.003**
Gestational weeks at birth	36.3±1.1	34.7±3.0	0.055
Emergency cesarean	5 (22.7)	6 (37.5)	0.291
Birth weight (gs)	2,844.1±303.0	2,660.7±838.8	0.389
APGAR fifth minute <7	1 (4.5)	1 (6.2)	0.811
NICU	3 (13.6)	6 (37.5)	0.074
Preterm delivery	10 (45.4)	12 (75)	0.069
Spontaneous	7 (31.8)	7 (43.7)	
Iatrogenic	3 (13.6)	5 (31.2)	
Neonatal pH*	7.47±0.2 (n=5)	7.29±0.1 (n=11)	**0.021**
Adverse obstetric outcome	10 (45.4)	12 (75)	0.069
Neonatal death	0 (0)	1 (6.2)	
Meconium delivery	0 (0)	1 (6.2)	

Note: Data are presented as mean±standard deviation, number (n), and percentage (%).

* Bold values denote statistical significance at the p<0.05 level. BA: bile acid; SuPAR: soluble urokinase plasminogen activator receptor; NICU: neonatal intensive care unit; ALT: alanine aminotransferase; AST: aspartate aminotransferase.

**Figure 1:**
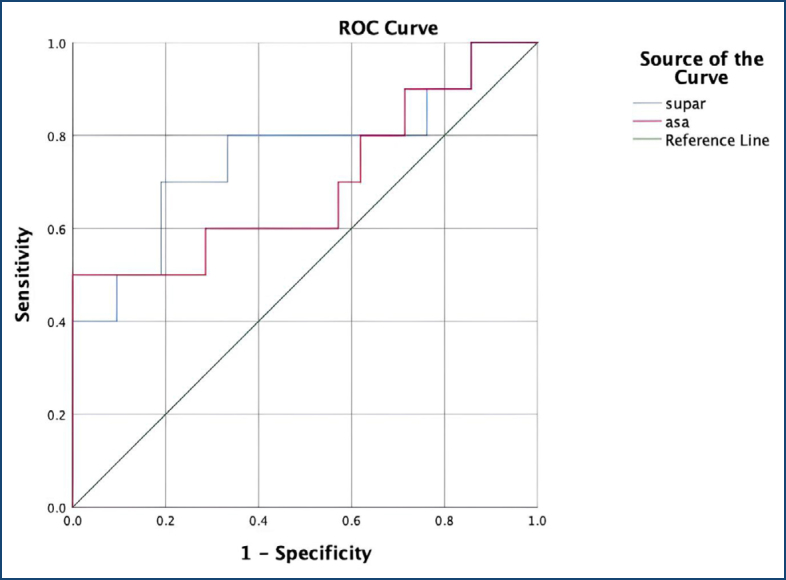
Receiver operating characteristic curve for soluble urokinase plasminogen activator receptor (blue line) and serum bile acid (red line) levels in predicting neonatal intensive care unit admission in the intrahepatic cholestasis of pregnancy group.

Figure 1 presents the ROC curve for the predictive performance of BA and suPAR levels in anticipating NICU admission within the ICP group. The discriminatory power of suPAR levels in predicting NICU need was found to be statistically significant (AUC: 0.757; 95%CI 0.552–0.962; p=0.022). For suPAR, the optimal cut-off point was calculated as 0.23 ng/mL with a sensitivity of 70.0% and specificity of 71.4% according to the Youden index. The maternal BA level yielded an AUC of 0.695 (95%CI 0.472–0.919; p=0.083). The optimal cut-off value for BA was identified as 40.4 μmol/L, with a sensitivity of 60.0% and specificity of 66.7% at this threshold.

## DISCUSSION

To our knowledge, this is the first study to investigate the relationship between ICP and suPAR levels. The system, consisting of an activator, an inhibitor, and a receptor, is associated with fibrinolysis, inflammation, and carcinogenesis. Although the reference ranges of suPAR levels in pregnancy have not been clearly established, suPAR has been previously studied in pregnancy complications such as preeclampsia, preterm birth, and chorioamnionitis.

There are differing opinions regarding maternal plasma suPAR levels and hypertensive disorders of pregnancy. Toldi et al.^11^ found that suPAR and inflammatory markers were elevated in preeclamptic women compared to the healthy group. Similarly, another small-scale study reported increased suPAR levels in women with severe preeclampsia^12^. In contrast to these findings, a retrospective study examining blood samples collected during antenatal screening before the onset of any symptoms in preeclamptic women found no association between suPAR levels and preeclampsia^13^.

In another study investigating maternal suPAR levels in relation to poor obstetric outcomes^14^, suPAR levels were found to be similar between pregnant women with preeclampsia, fetal growth restriction, and healthy controls. However, in the group with spontaneous preterm labor, suPAR levels were 3.1 ng/mL (2.3–5.0), compared to 2.6 ng/mL (0.9–6.2) in the healthy control group. SuPAR was also investigated in amniotic fluid in pregnant women. In patients with preterm premature rupture of membranes, amniotic fluid suPAR levels were found to be high in the presence of fetal inflammation and histologic chorioamnionitis^15,16^. In this study, we found elevated maternal suPAR levels in ICP, along with a statistically significant correlation with NICU admission. However, the suPAR levels were lower than those reported in previous studies. We think that this may be due to the difference in the kit used. In a study, suPAR measurements obtained by a conventional ELISA kit were compared with those from a turbidimetric immunoassay kit. It was reported that suPAR levels measured by the conventional ELISA method were approximately 40% lower than those measured by the turbidimetric immunoassay^17^. This difference highlights the impact of the assay methodology on suPAR measurement. We would also like to point out that there is still no clear value of suPAR level in pregnancy. In addition, there are studies showing that suPAR levels decrease as pregnancy progresses. Our study included the late ICP group. This may have affected suPAR values. SuPAR has been studied in previous research in diseases associated with higher mortality and greater inflammation compared to ICP, such as COVID-19, intensive care admissions, or cancer^18^. SuPAR is also cleared from circulation through renal excretion and cardiac clearance^19^. Among our pregnant patients, there is no condition that would affect suPAR clearance. These factors may also explain why our current findings are lower compared to other studies.

In previous studies, high suPAR levels have been associated with prognosis and survival in adult liver diseases. In patients with liver inflammation or fibrosis, elevated suPAR levels were shown to indicate progression to hepatocellular carcinoma and were demonstrated to have prognostic significance^20^. In patients who developed liver failure due to causes such as autoimmune conditions or drug toxicity, suPAR was found to be higher compared to healthy people, and they were also considered significant in predicting the need for transplantation and mortality^21^. Interestingly, another study reported that individuals with a healthy lifestyle had lower suPAR, and those with low suPAR were found to have better 5-year survival rates^22^.

ICP is one of the common hepatic disorders in pregnancy, known for its adverse obstetric and neonatal outcomes. Recent evidence suggests that elevated BA levels in ICP may be linked to maternal endothelial dysfunction, possibly through mechanisms involving oxidative stress and vascular inflammation^23^. Furthermore, alterations in vascular endothelial growth factors observed in pregnancies complicated by ICP suggest that endothelial cells may be adversely affected in this condition^24^. These findings support the notion that ICP is not solely a hepatic disorder but may also involve systemic endothelial dysfunction. In this context, investigating inflammatory biomarkers such as suPAR gains pathophysiological relevance in evaluating the prognostic landscape of ICP. In the current literature, BA levels play a diagnostic role, with levels above 40 associated with increased ICP-related complications and levels above 100 linked to fetal death^25^. Due to such complications, outcomes for both mothers with ICP and their offspring are of significant concern. In our study, we demonstrated that elevated suPAR levels reflect the inflammatory nature of ICP and that this marker may be useful in predicting NICU admission. However, it should be considered that the moderate discriminatory power of suPAR may not be sufficient alone for clinical decision-making.

## CONCLUSION

Maternal plasma suPAR levels were found to be elevated in ICP. Elevated maternal suPAR levels may help predict adverse pregnancy outcomes.

## Data Availability

The datasets generated and/or analyzed during the current study are available from the corresponding author upon reasonable request.
